# A Versatile Equilibrium Method for the Synthesis of High-Strength, Ladder-like Polyphenylsilsesquioxanes with Finely Tunable Molecular Parameters

**DOI:** 10.3390/polym13244452

**Published:** 2021-12-18

**Authors:** Tatyana O. Ershova, Anton A. Anisimov, Maxim N. Temnikov, Maxim A. Novikov, Mikhail I. Buzin, Galina G. Nikiforova, Yulia S. Dyuzhikova, Ivan E. Ushakov, Olga I. Shchegolikhina, Aziz M. Muzafarov

**Affiliations:** 1 A. N. Nesmeyanov Institute of Organoelement Compounds, Russian Academy of Sciences, 119991 Moscow, Russia; ershovatatyana_2995@mail.ru (T.O.E.); buzin@ineos.ac.ru (M.I.B.); ggn@ineos.ac.ru (G.G.N.); vysochinskaja@yandex.ru (Y.S.D.); f0rbmen@gmail.com (I.E.U.); olga@ineos.ac.ru (O.I.S.); aziz@ispm.ru (A.M.M.); 2 Zelinsky Institute of Organic Chemistry, Russian Academy of Sciences, 47 Leninsky Pr., 119991 Moscow, Russia; manovikov@ioc.ac.ru; 3 N. S. Enikolopov Institute of Synthetic Polymeric Materials, Russian Academy of Sciences, 117393 Moscow, Russia

**Keywords:** ladder-like polyphenylsilsesquioxanes, ammonia, condensation

## Abstract

A versatile equilibrium method for synthesizing ladder-like polyphenylsilsesquioxanes (L-PPSQs) with various molecular weights (from 4 to 500 kDa) in liquid ammonia was developed. The effect of diverse parameters, such as temperature, monomer concentration, reaction time, addition or removal of water from the reaction medium, on the polycondensation process was determined. The molecular weight characteristics and structure of the L-PPSQ elements obtained were determined by GPC, ^1^H, ^29^Si NMR, IR spectroscopy, viscometry, and PXRD methods. The physicochemical properties of L-PPSQs were determined by TGA and mechanical analyses.

## 1. Introduction

Polyphenylsilsesquioxanes (PPSQs) are important organosiloxanes with unique physicochemical properties, for instance, high thermal and radiation resistance, high refractive index, mechanical properties, and solubility in organic solvents such as chloroform, benzene, toluene, dichlorobenzene, xylene, and tetrahydrofuran (THF) [1,2]. Due to these properties, they are widely used as protective, dielectric, hydrophobic, heat-resistant coatings, modifiers of organic and organoelement formulations [3,4,5,6,7,8], and materials for optoelectronics [9,10].

The regular structure is yet another feature of PPSQs. PPSQs are usually obtained by polycondensation of phenyltrichloro- or -trialkoxysilanes. The reaction conditions determine the final structure of the PPSQ molecule [11]. As a result, it is possible to obtain PPSQs whose molecules have polyhedral, hyperbranched, statistical, or ladder-like structures.

Ladder-like PPSQs were first described by Brown et al. in 1960 [12], but their synthesis and study of their properties have continued to attract the attention of scientists for many decades. Such polymers having a molecular weight in the range of 10^6^ Da can form strong transparent films, with a high refractive index, and may find application as promising polymer matrices, membranes, materials for waveguides, etc. [3,6,13,14,15,16,17,18,19].

There are several approaches to the synthesis of L-PPSQs. The first approach involves the high-temperature (250 °C) polymerization of phenyltrichlorosilane hydrolysis products in the presence of a strong base [1]. Soviet scientists also performed studies in this area. They identified various factors that affect the molecular weight and structural characteristics of the resulting polymers [20,21]. An important result was obtained in a study that demonstrated the reversible nature of the polymerization reaction and the stability of the most thermodynamically stable form of polyhedral octaphenylsilsesquioxane. The search for more efficient alternative approaches for synthesizing L-PPSQs continues. For example, Zhang et.al. [22] suggested a method that involves the condensation of a preorganized 1,1,3,3-tetraphenyldisiloxane-1,3-diol monomer. This method assumes mild synthesis conditions; however, it is a multistage method similar to that described above. It should also be noted that the L-PPSQ elements obtained had relatively low molecular weights (up to 2 × 10^3^ Da). This approach was developed further [23,24] using ethylenediamine as a template for the preorganization of PPSQs. After hydrolysis of this structure, L-PPSQs with M_w_ = 5 × 10^4^ Da were formed. Choi [25] suggested the simplest method for synthesizing L-PPSQs that involved the hydrolysis of phenyltrimethoxysilane in the presence of K_2_CO_3_. The key factor in this reaction is the concentration of the initial monomer. In fact, T10 polyhedral decamer was formed at low monomer concentrations, while L-PPSQs with M_W_ up to 1.5 × 10^4^ Da were formed at high concentrations. It can be observed from the above data that a versatile directed method for the synthesis of L-PPSQs with a predetermined molecular weight (M_W_) has not been found to date.

In recent decades, there has been a growing interest in compressed gas media as new solvents for chemical reactions, including condensed gases and supercritical fluids [26,27,28,29]. The main advantages of these media include the possibility of adjusting their dissolving capacity and their instant removal from the reaction media. The latter feature is most important since the product obtained in this way does not require expenses for purification from the solvent.

Earlier, we developed a new method for the synthesis of phenyl-containing siloxanes in ammonia media. The advantage of this method is that ammonia plays the role of both a solvent and a catalyst. At the same time, the reaction products do not require purification, since ammonia is removed from the reaction medium and can be reused in the future. We have shown that phenylcyclosiloxanes, disiloxanes, and L-PPSQs with a molecular weight of M_W_ = 164 kDa can be obtained in an ammonia medium [30,31]. This method is promising because it employs an active medium—namely, ammonia whose properties can be altered in a wide range by varying the external conditions: pressure, temperature, and concentration of initial reagents.

Ammonia is a large-scale product from the chemical industry [32] that can be applied in various fields: fertilizers, refrigeration, medicine, etc. [33,34]. It should also be noted that ammonia can be used in organic and inorganic synthesis as a reagent and solvent [35,36,37,38]. Therefore, it is actively used in the synthesis of amides and amines [39]. It is worth noting separately that ammonia is used in the treatment and modification of polymers [37]. In organosilicon chemistry, ammonia is a key reagent in the synthesis of silazanes, an important class of organosilicon compounds [40].

This article presents the results of our studies on the effect of various factors on the condensation of *cis*-tetraphenylcyclotetrasiloxanetetraol (*cis*-tetraol) in ammonia. The structure and molecular weight characteristics of the resulting L-PPSQs, as well as physicomechanical properties of the obtained samples, allow us to state that the process is versatile in terms of controlling the structure and properties of the reaction products.

## 2. Results and Discussion

### 2.1. Synthesis

**Figure 1:**
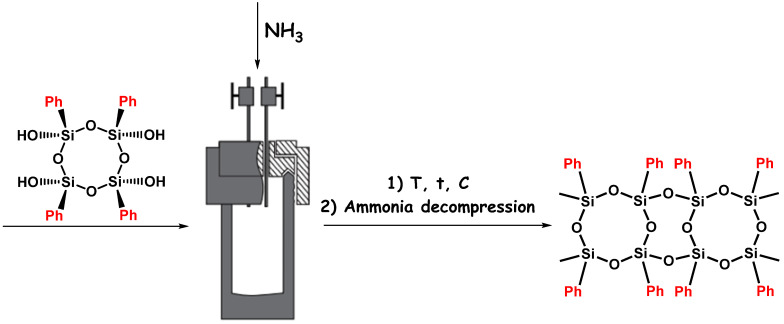
General scheme of L-PPSQ synthesis in ammonia medium.

The condensation of *cis*-tetraphenylcyclotetrasiloxanetetraol (*cis*-tetraol) was performed in high-pressure steel reactors (see Figure 1).

A certain amount of *cis*-tetraol was loaded into a high-pressure reactor, which was then cooled to −50 °C, and ammonia was pumped using a flow controller. Then, the reactor was immersed into a thermostatic bath at a given temperature. After a certain time, the reactor was cooled to room temperature, ammonia was decompressed, and the polymer was isolated.

**Figure 2:**
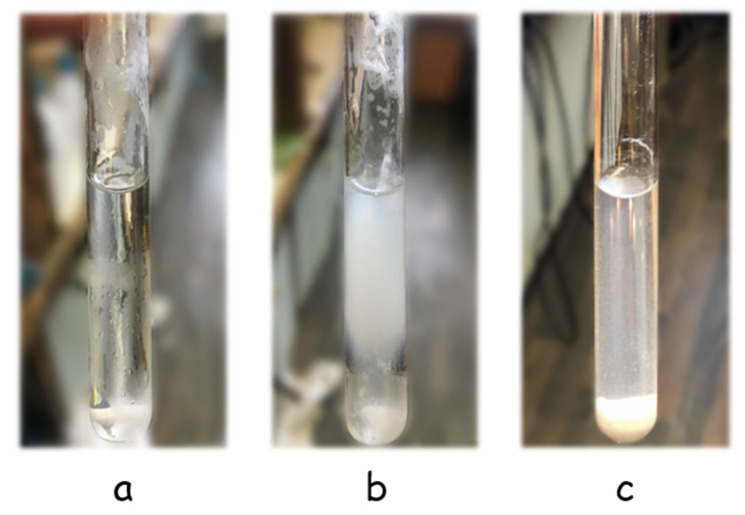
(**a**) Solubility of the monomer in ammonia, (**b**) 15 min after dissolution, and (**c**) 1 h after dissolution.

To assess the solubility of the initial monomer and visualize the reaction, we carried out an experiment in test tubes at room temperature (see Figure 2). As can be seen from Figure 2a, the monomer dissolves in ammonia to form an ideal solution. The condensation products begin to precipitate from the solution after 15 min (Figure 2b). The polymer precipitated as a separate phase can be distinctly observed an hour later (Figure 2c).

The reaction products were analyzed by GPC, NMR, IR spectroscopy, PXRD, and viscometry in solution.

### 2.2. Monomer Concentration Effect

**Figure 3:**
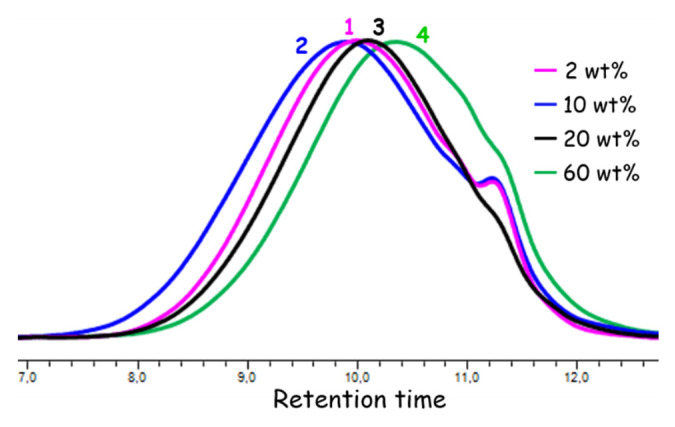
GPC curves obtained in Experiments **1**–**4.**

The effect of monomer concentration on the condensation process was studied at a temperature of 30 °C using a reaction time of 4 h (Figure 3, Table 1, Experiments **1**–**4**).

It can be observed from the data presented in Table 1 (Experiments **1**–**4**) that the monomer concentration does not significantly affect the molecular weight characteristics of the polymers obtained. It can be concluded from the above data that 10 wt% is the optimal concentration of the monomer to produce a polymer with a higher molecular weight under these reaction conditions. It should also be noted that a decrease in the monomer concentration below 20 wt% results in low molecular weight products, as it can be observed from the bimodal nature of the curves obtained in experiments **1** and **2**. It can be assumed that polyhedral compounds containing few hydroxy groups are formed in this case. The effect of monomer concentration on the structure of the products was also previously [24].

### 2.3. Reaction Time Effect

**Figure 4:**
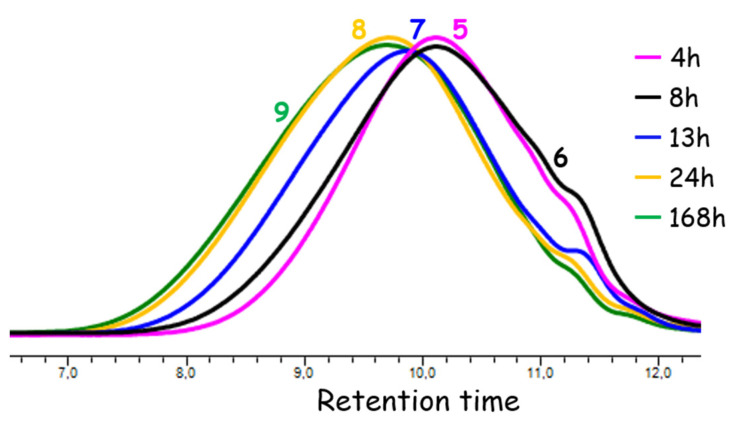
GPC curves obtained in Experiments **5**–**9.**

The effect of reaction time on the condensation process was studied at 30 °C and a monomer concentration of 20 wt% (see Figure 4, Table 1, Experiments **5**–**9**). The reaction was performed for 4 to 168 h.

**Figure 5:**
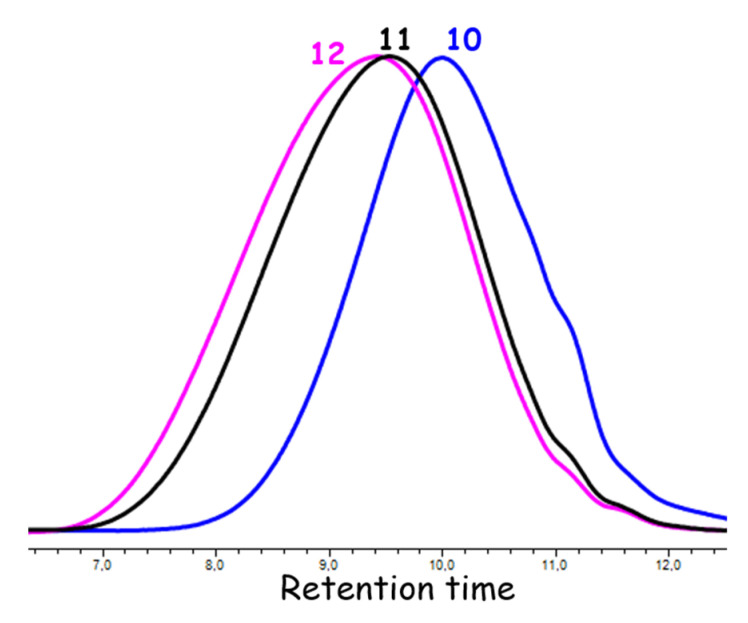
GPC curves obtained in experiments **10**—L-PPSQ synthesis; **11**—synthesis after first decompression; **12**—synthesis after second decompression.

It can be observed from GPC data that the molecular weight of condensation products ceases to increase after 24 h. Probably, the polycondensation reaction reaches dynamic equilibrium due to the release of water in the condensation of *cis*-tetraol in the first stage. After the release of water, M_W_ growth occurs by the polymerization–condensation mechanism. The growth of M_W_ stops upon reaching an equilibrium. To confirm this assumption, we carried out experiments using the “condensation–decompression–condensation” consequent cycle. In this case, water resulting from the reaction is removed along with ammonia from the reactor. It can be observed from Figure 5 that after the second condensation (Experiment **12**), the molecular weight ceases to increase significantly in comparison with Experiment **11** (Table 1, Experiments **10**–**12**), i.e., the condensation processes naturally slow down as the concentrations of reacting groups decrease.

The reaction time does not significantly affect the molecular weight characteristics of L-PPSQs, as it can be observed from the data obtained.

### 2.4. Temperature Effect

**Figure 6:**
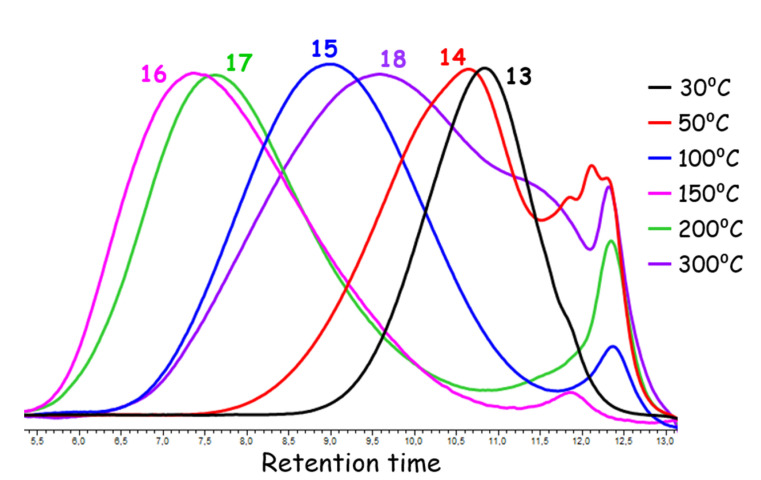
GPC curves obtained in Experiments **13**–**18**.

The condensation of *cis*-tetraol was performed at temperatures from 30 to 300 °C for 4 h at 20% monomer concentration (Figure 6, Table 1, Experiments **13**–**18**).

The temperature of *cis*-tetraol condensation in ammonia has a significant effect on this process as can be seen from the data obtained. As the temperature rises from 30 to 150 °C, the M_W_ of the product increases. The largest M_W_ of the polymer was obtained at 150 °C (**16**). A further increase in temperature results in a decrease in the M_W_ of the reaction products. This is due to the fact that the equilibrium is shifted towards the formation of low-molecular-weight products at temperatures of 200 and 300 °C, which agrees with the data published earlier [41,42].

Thus, we showed for the first time that it is possible to obtain ladder polyphenylsilsesquioxane with a certain molecular weight in the range of 8–500 kDa. To this end, it is sufficient to vary the reaction temperature within 30–150 °C. A further increase in temperature activates depolymerization processes, and hence, the M_W_ decreases.

### 2.5. Water Effect

**Figure 7:**
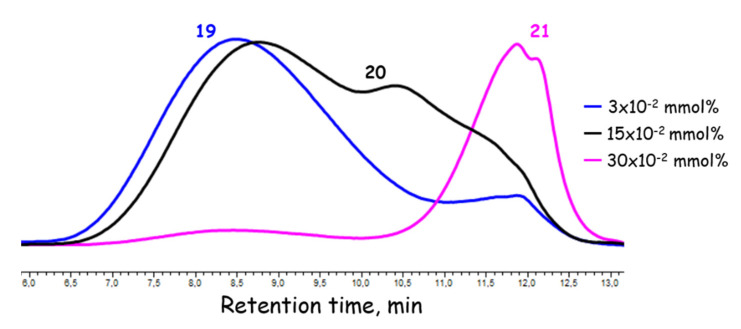
GPC curves obtained in experiments **19**–**21**.

It appears that the presence of water in the system is an important factor affecting the condensation of *cis*-tetraol in ammonia. Water is formed upon homofunctional condensation of silanol groups and limits the growth of L-PPSQ molecular weight, as shown above. Its removal leads to a shift of equilibrium and an increase in the M_W_ of the resulting polymer. We found that an additional amount of water in the system also affects the polycondensation process. Experiments with the addition of 3 × 10^−2^, 15 × 10^−2,^ and 30 × 10^−2^ mmol% water (Experiments **19**–**21**) into the reaction system were performed. An increase in the water amount results in the formation of polymers with a lower molecular weight, as can be observed from the data shown in Figure 7 and Table 2.

**Figure 8:**
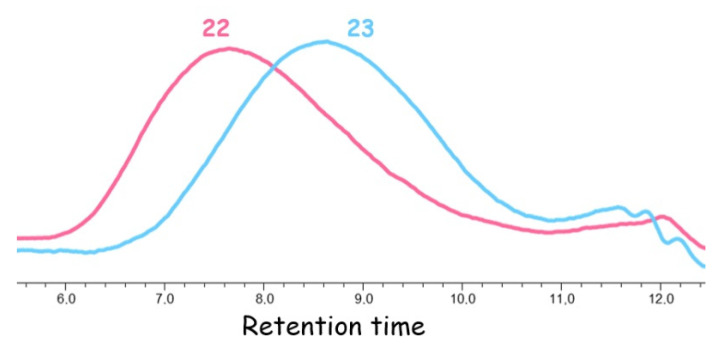
GPC curves obtained in Experiments **22**—L-PPSQ with silanol groups blocked by trimethylchlorosilane; **23**—condensation product of sample **22** with addition of water.

It is likely that this effect may be associated with the reaction mechanism. We assume that the mechanism of L-PPSQ formation in the system in question is largely similar to the mechanism of high-temperature polymerization first suggested by Brown and subsequently studied in detail by Soviet scientists [41]. It involves the anionic polymerization of phenyltrichlorosilane hydrolysis products, where potassium hydroxide acts as the initiating agent. Accordingly, an increase in its amount should lead to a decrease in the product’s M_W_ and an increase in the fraction of side processes (depolymerization, chain transfer). In our case, the addition of more water results in the formation of a larger amount of NH_4_OH in the system. This compound may have an effect similar to potassium hydroxide under liquid ammonia conditions. On the other hand, water is also released in the homocondensation of silanol groups. In this case, its additional amount displaces the equilibrium towards the formation of low-molecular products. This assumption was proved by the following experiment. At first, the residual silanol groups in the L-PPSQ sample were blocked with trimethylchlorosilane, then its reaction with water was carried out. It can be observed from Figure 8 that in this case, the siloxane bond is broken and a product with a smaller molecular weight is formed.

It can be observed from Table 3 that the molecular weight of the L-PPSQ sample decreases twofold.

**Figure 9:**
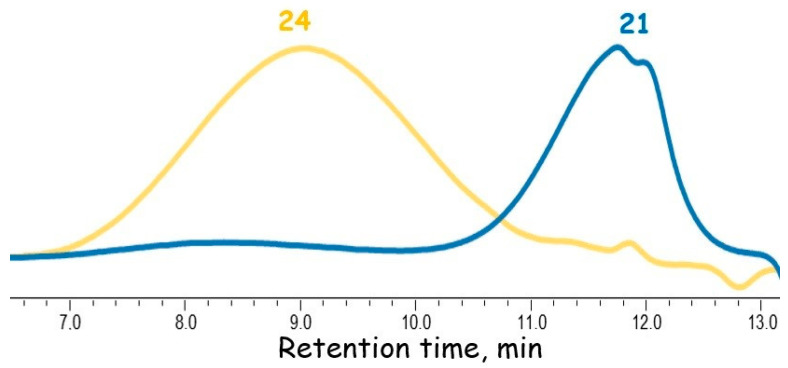
GPC curves obtained in Experiment **21** is the low molecular weight condensation product of *cis*-tetraol with addition of water; Experiment **24** is the product of condensation of sample **21**.

It should be noted that if the low-molecular-weight product obtained in Experiment **21** is subjected to the polymerization reaction again without water addition, a high-molecular L-PPSQ can be obtained (see Figure 9, Table 3). In other words, the reaction is completely reversible and this fact emphasizes its versatility. By adjusting the process parameters, it is possible to synthesize products with certain molecular parameters and also to convert the polymer into the starting compounds for reuse.

### 2.6. Isolation and Study of L-PPSQ Structure

**Scheme 1:**
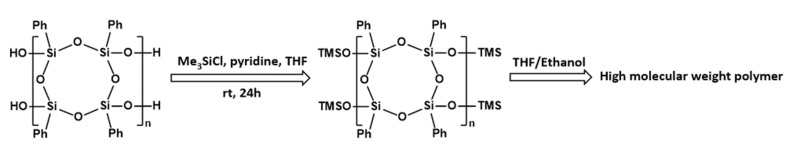
Blocking of residual silanol groups in L-PPSQ and followed by reprecipitation of the resulting polymers.

To determine the structure and properties of the L-PPSQ samples obtained, we blocked the residual silanol groups with trimethylchlorosilane in the presence of pyridine. After that, the products were reprecipitated in the ethanol/THF system (Scheme 1) and analyzed.

**Figure 10:**
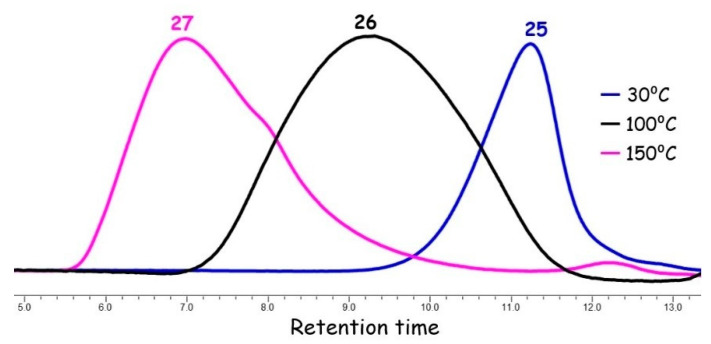
GPC curves obtained in Experiments **25**–**27**.

In this way, three samples of polymers synthesized at 30, 100, and 150 °C were prepared (**25**–**27**). Their molecular weight characteristics are shown in Figure 10 and in Table 4.

It is known that L-PPSQ samples obtained by the classic high-temperature method have a defective structure [43,44], i.e., the content of SiOH groups in the polymers exceeds the theoretical value. The ratio of the measured and calculated values of the content of hydroxy groups can provide information about the defectiveness of the polymers obtained. It can be seen from the data in Table 4 that the defectiveness of the structure of the synthesized polymers increases in proportion to the molecular weight. At the same time, this value of sample **25** obtained under the mildest conditions is close to the theoretical one. The content of SiOH groups regularly decreases with an increase in the MW of samples, which indicates its predominantly linear-ladder structure. The results obtained from the analysis of functional groups allow us to use L-PPSQ samples as accessible blocks for synthesizing block copolymers as an alternative to linear/ladder-like polysiloxane block copolymers used previously [7,45,46].

**Figure 11:**
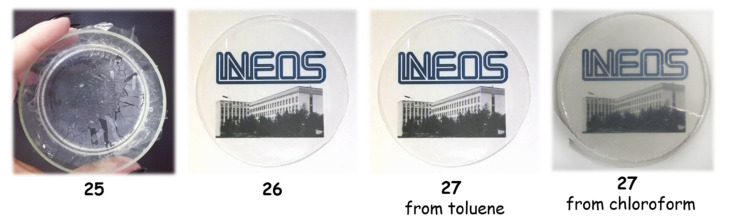
Polymer films **25**–**27.**

The three polymer samples isolated by this method were used to obtain films from 1% solutions in toluene by casting onto cellophane support. PPSQ samples obtained at 100 °C (**26**) and 150 °C (**27**) form hard transparent films, whereas the polymer sample with a lower molecular weight obtained at 30 °C (**25**) is unable to form continuous films (see Figure 11).

The ladder structure of polymers was confirmed by a set of physicochemical methods—namely, NMR, IR spectroscopy, and PXRD.

### 2.7. NMR and IR Spectroscopy

The L-PPSQ samples synthesized (**25**–**27**) were studied by NMR and IR spectroscopy.

^1^H NMR spectra contain two main peaks at 6.2–7.8 ppm and −0.67–0.08 ppm belonging to the PhSiO_1.5_ and terminal Me_3_SiO_0.5_ groups, respectively. Their ^29^Si NMR spectra exhibit two peaks corresponding to PhSiO_1.5_ groups (−80 ppm) and Me_3_SiO_0.5_ terminal groups (10 ppm).

**Figure 12:**
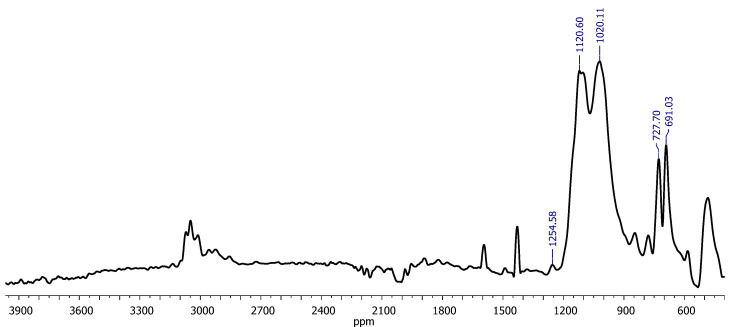
IR spectra of L-PPSQ **27**.

The IR spectra of the L-PPSQ samples obtained show absorption bands typical of phenyl groups (691, 727 cm^−1^), Si-O-Si bonds (1020–1120 cm^−1^), and Me_3_SiO_0.5_ groups 1254 cm^−1^). There are no stretching bands typical of –SiOH groups in the region of 3100–3500 cm^−1^. This fact confirms that silanol groups are completely blocked with trimethylchlorosilane (see Figure 12).

The NMR and IR spectroscopy data obtained agree with the results previously published for L-PPSQ samples [47].

### 2.8. Powder X-ray Diffraction Analysis (PXRD)

**Figure 13:**
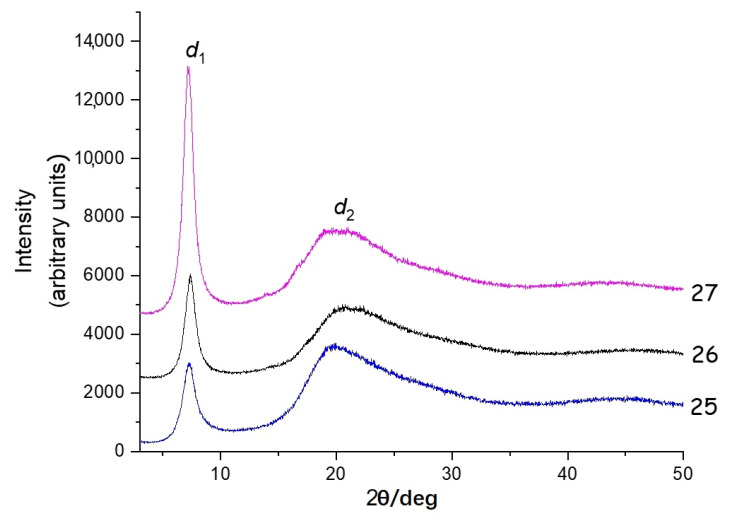
Diffraction data for polymers **25**–**27**.

Powder X-ray phase analysis (XRD) for samples **25**–**27** shows two distinct diffraction halos, in good agreement with previous works. The first halo at 7.2–7.3° (d_1_), indicating the intramolecular chain-to-chain distance (i.e., the width of each double chain) in the double-chained, ladder-like molecule is narrow and sharp. It is indicated that L-PPSQs have a sufficiently rigid skeleton in which there is limited movement around the longitudinal axis, and the conformation is practically invariable. The second diffuse halo indicates that the average thickness of the ladder-like polymer chain is 19.7–19.8° (d_2_) (see Figure 13, Table 5). These values agree with the results described in the literature for L-PPSQs [48].

### 2.9. Viscosity Measurements

**Figure 14:**
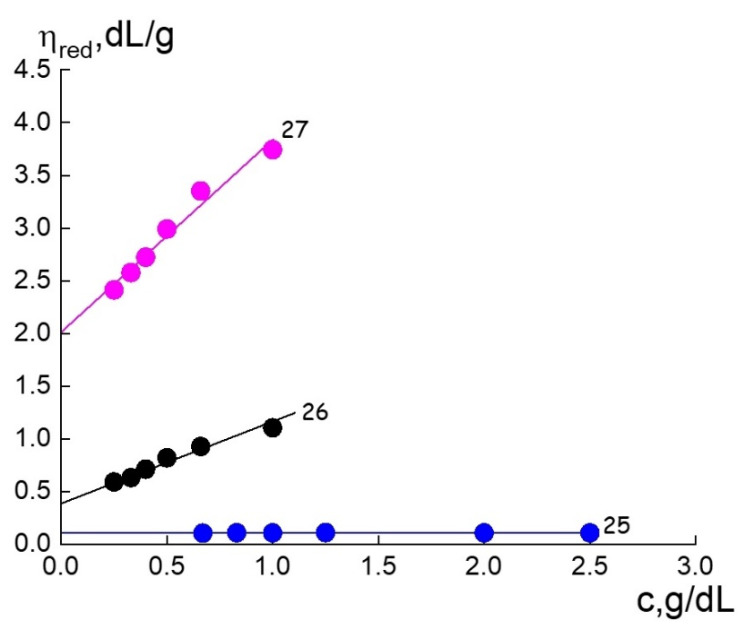
Reduced viscosity vs. concentration for dilute solutions of polymers **25**–**27** in toluene (T = 37 °C).

Figure 14 and Table 6 show the values of the intrinsic viscosity of solutions of the polymers in toluene at 37 °C and their molecular weights Mn calculated by the formula [η] = 1.77 × 10^−5^ × M^0.895^. The formula was derived from a study of 18 molecular weights (0.26–4.88) × 10^5^ Mn of polymer fractions^1^ and is applicable in a wide range of molecular weights.

It is worth noting that the reduced viscosity of polymer **25** nearly does not depend on the concentration of the solutions studied. This dependence of the viscosity of L-PPSQ solutions on M_W_ coincides with literature data [49,50].

## 3. Exploration of L-PPSQ Properties

### 3.1. Thermal Characteristics

**Figure 15:**
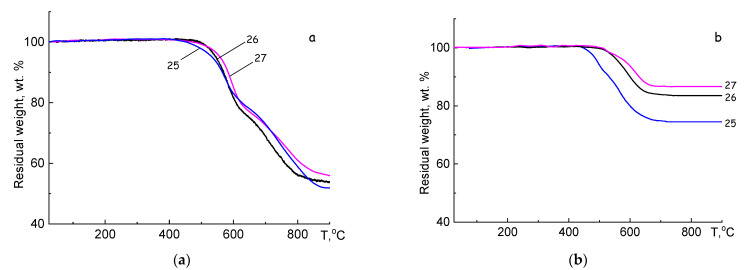
TGA curves of polymers **25**–**27** obtained at a heating rate of 10 degrees/min: (**a**) in air; (**b**) in argon.

L-PPSQ samples were studied by the TGA method in air and in argon. The results obtained are shown in Figure 15a,b and Table 7. The decomposition onset temperature of the samples synthesized and the amount of solid residue after the end of thermal transformations are in good agreement with literature data [51,52]. The onset temperature of polymer samples grows with an increase in their molecular weight, both in air and in argon. While the percentage of solid residue in the air atmosphere for all L-PPSQ samples differs insignificantly (Figure 15a, Table 7), the percentage of solid residue in the argon atmosphere changes appreciably (Figure 15b, Table 7) symbatically with an increase in the molecular weight. This effect may be due to the specifics of the elimination and decomposition of phenyl groups that are responsible for the carbonization of the solid residue of L-PPSQ with different molecular weights.

It should be noted that the thermal characteristics obtained for polymer **27** are superior to the results published previously [53].

### 3.2. Physical and Mechanical Measurements

The mechanical properties of polymers obtained at 100 and 150 °C (**26** and **27**) were studied by the uniaxial extension method.

**Figure 16:**
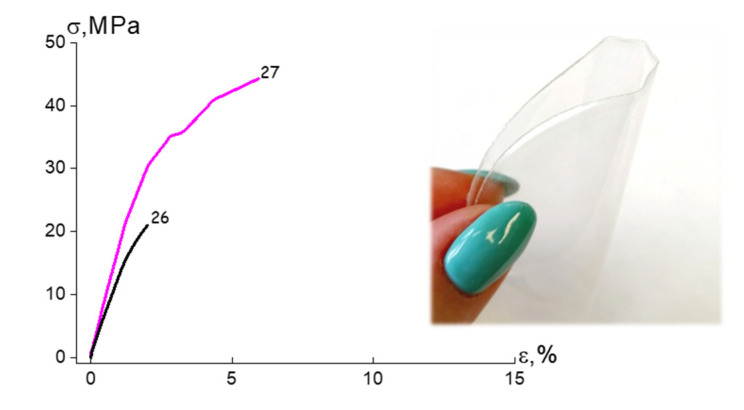
Stretching curves of polymers **26**–**27**.

A film obtained from the polymer synthesized at 150 °C that had the largest molecular weight (**27**) (see Figure 16, Table 8) showed the best mechanical characteristics. The values of ultimate tensile stress (σ) and elongation at break (ε) found for polymer **27** match the best characteristics of L-PPSQs reported in the recent literature [53].

Curve **26** is almost linear and corresponds to Hooke’s deformation. This suggests that there is nearly no segmental mobility or mobility of macromolecules as a whole, which are responsible for elasticity and plasticity, respectively, in this polymer. At the same time, sample **27** behaves somewhat differently as it can be observed from the deviation of the tensile curve from Hooke’s plot. It can be ascribed to the greater defectivity of sample **27** (Table 4). Defects can disrupt the structure of the double-stranded chain. So-called hinges, i.e., places where rotation around the siloxane bond is possible, appear in it. It is likely that segmental mobility appears in a molecule with a sufficient number of such hinges. As a result, the ultimate deformation of sample **27** increases to 6%, which is three times larger than that of sample **26**.

## 4. Conclusions

A versatile method for the synthesis of L-PPSQs with controlled molecular weight characteristics was developed for the first time. The effect of various parameters (temperature, monomer concentration, reaction time, addition, and removal of water to/from the reaction medium) on the polycondensation process was studied. It was found that the reaction temperature and amount of water in the system are the main parameters determining the molecular weight characteristics of L-PPSQs. The molecular weight characteristics and structures of the polymers were confirmed by GPC, ^1^H, ^29^Si NMR, IR spectroscopy, viscosity measurements, and PXRD. The high-molecular-weight L-PPSQs obtained are capable of forming flexible transparent films with high mechanical and thermal characteristics. The important factors of the new process include the possibility to produce functional blocks with various molecular weights and high-strength samples with a very promising set of properties for critical practical applications. These processing characteristics, along with the previously shown [29] possibility of complete recycling of the reaction medium, i.e., ammonia, indicate their full compliance with the modern requirements for polymer materials and processes of their production within the framework of the sustainable development concept.

## Figures and Tables

**Table 1:** Experimental data on the effect of monomer concentration, reaction time, and temperature.

Experiment	Monomer Concentration, wt%	Temperature, °C	Reaction Time, h	Mn, kDa	Mw, kDa	Mp, kDa	Mw/Mn
**1**	2	30	4	4.6	9.1	6.1	1.9
**2**	10	30	4	5.1	11.1	6.8	2.2
**3**	20	30	4	4.2	8.1	5.1	1.9
**4**	60	30	4	3.8	7.1	4.0	1.9
**5**	20	30	4	4.2	8.1	5.1	1.9
**6**	20	30	8	5.1	10.4	6.3	2.0
**7**	20	30	13	5.8	12.7	7.8	2.2
**8**	20	30	24	6.4	14.5	8.5	2.3
**9**	20	30	168	6.3	14.9	8.7	2.4
**10**	20	30	4	4.0	7.6	5.1	1.9
**11**	-	30	4	8.2	18.2	10.6	2.2
**12**	-	30	4	9.4	22.1	12.0	2.4
**13**	20	30	4	7.8	15.5	9.4	1.9
**14**	20	50	4	14.4	34.1	14.7	2.4
**15**	20	100	4	60.1	144.1	95.3	2.4
**16**	20	150	4	189.9	477.9	461.7	2.5
**17**	20	200	4	145.5	461.6	361.6	3.2
**18**	20	300	4	13.0	95.2	56.4	7.3

**Table 2:** Data of experiments on the water effect.

Experiment	Monomer Concentration, wt%	H_2_O Concentration, mmol%	Temperature, °C	Reaction Time, h	HMWF ^[a]^		Mw/Mn	LMWF ^[b]^		Mw/Mn
					Mn, kDa	Mw, kDa		Mn, kDa	Mw, kDa	
**19**	20	3 × 10^−2^	150	4	51.4	134.1	2.6	0.8	2.0	2.6
**20**	20	15 × 10^−2^	150	4	72.3	120.7	1.7	2.2	8.6	3.8
**21**	20	30 × 10^−2^	150	4	95.5	150.0	1.6	1.2	2.9	2.5

^[a]^ HMWF—high molecular weight fraction; ^[b]^ LMWF—low molecular weight fraction.

**Table 3:** Data from experiments **21**–**24** with water.

Experiment	Monomer Concentration, wt%	H_2_O Concentration, mmol%	Temperature, °C	Reaction Time, h	Mn, kDa	Mw, kDa	Mp, kDa	Mw/Mn
**22**	20	-	150	4	102.7	283.2	276.5	2.8
**23**	-	30 × 10^−2^	150	4	54.4	136.1	110.2	2.5
**21**	20	30 × 10^−2^	150	4	1.2	2.9	1.5	2.45
**24**	-	-	150	4	16.9	61.7	46.8	3.6

**Table 4:** Molecular weight characteristics of polymers **25**–**27**.

Experiment	Monomer Concentration, wt%	Temperature, °C	Reaction Time, h	Mp, kDa	Mw, kDa	Mn, kDa	Mw/Mn	Wt OH, %w	WnmrOH, %w	Wt/W nmr
**25**	20	30	4	14.2	22.4	12.3	1.8	0.550	0.62	1.1
**26**	20	100	4	116.6	161.9	67.2	2.4	0.100	0.14	1.4
**27**	20	150	4	558.4	549.2	196.8	2.4	0.035	0.07	1.9

**Table 5:** Values of PXRD parameters for polymer samples **25**–**27**.

Experiment	d_1_, nm	d_2_, nm
**25**	12.2	4.5
**26**	12.3	4.4
**27**	12.2	4.5

**Table 6:** Molecular weight characteristics of polymers **25**–**27**.

Experiment	Mw, kDa	Mn, kDa	Mη, kDa	[η], dL/g
**25**	22.4	12.3	15.6	0.1
**26**	161.9	67.2	73.3	0.4
**27**	549.2	196.8	442.5	2.2

**Table 7:** Thermal data of polymers **25**–**27**.

Experiment	T_d_^5%^,°C Air	M_res_, wt% Air	T_d_^5%^, °C Argon	M_res_, wt% Argon
**25**	527	52	464	75
**26**	536	54	557	85
**27**	537	56	587	87

**Table 8:** Mechanical characteristics of polymers **26**–**27**.

Experiment	E, MPa	σ _p_, MPa	ε_p_, %
**26**	1300	21	2
**27**	1700	44	6

## Data Availability

Not Applicable.
